# A computational fluid dynamics investigation of endothelial cell damage from glaucoma drainage devices

**DOI:** 10.1038/s41598-023-50491-9

**Published:** 2024-02-15

**Authors:** Nicol Basson, Chao-Hong Surachai Peng, Patrick Geoghegan, Tshilidzi van der Lecq, David Steven, Susan Williams, An Eng Lim, Wei Hua Ho

**Affiliations:** 1 Department of Thermal & Fluid Engineering, Faculty of Engineering Technology, University of Twente, Enschede, 7522 NB The Netherlands; 2 Mechanical, Industrial & Aeronautical Engineering, Faculty of Engineering & the Built Environment, University of Witwatersrand, Johannesburg, Private Bag 3, Johannesburg, Wits, 2050 South Africa; 3 Department of Mechanical, Biomedical and Design Engineering, School of Engineering & Technology, College of Engineering and Physical Sciences, Aston University, Birmingham, B4 7ET UK; 4 Division of Ophthalmology, Faculty of Health Sciences, University of Cape Town, Cape Town, South Africa; 5 Division of Ophthalmology, School of Clinical Medicine, Faculty of Health Sciences, University of the Witwatersrand, Johannesburg, South Africa; 6 Bioprocessing Technology Institute, Agency for Science, Technology and Research, 20 Biopolis Way, #06-01 Centros, Singapore, 138668 Singapore; 7 School of Mechanical and Aerospace Engineering, Nanyang Technological University, 50 Nanyang Avenue, Singapore, 639798 Singapore

**Keywords:** Biomedical engineering, Computational science

## Abstract

Glaucoma drainage devices (GDDs) are prosthetic-treatment devices for treating primary open-angle glaucoma. Despite their effectiveness in reducing intraocular pressures (IOP), endothelial cell damage (ECD) is a commonly known side-effect. There have been different hypotheses regarding the reasons for ECD with one being an induced increase in shear on the corneal wall. A computational fluid dynamics (CFD) model was used to investigate this hypothesis in silico. The Ahmed Glaucoma Valve (AGV) was selected as the subject of this study using an idealised 3D model of the anterior chamber with insertion angles and positions that are commonly used in clinical practice. It was found that a tube-cornea distance of 1.27 mm or greater does not result in a wall shear stress (WSS) above the limit where ECD could occur. Similarly, a tube-cornea angle of 45° or more was shown to be preferable. It was also found that the ECD region has an irregular shape, and the aqueous humour flow fluctuates at certain insertion angles and positions. This study shows that pathological amounts of WSS may occur as a result of certain GDD placements. Hence, it is imperative to consider the associated fluid force interactions when performing the GDD insertion procedure.

## Introduction

The inside of the eye is divided into three sections or chambers namely the anterior, posterior and vitreous chambers. The anterior chamber is at the front of the eye, vitreous chamber at the back and the posterior chamber sandwiched in between. Biologically, the anterior chamber is the fluid-filled space located between the cornea and the iris and is filled with a clear, watery fluid called aqueous humour (AH), which helps maintain the shape of the eye and provides nutrients to the cornea and lens. The posterior chamber is between the iris and the lens and the vitreous chamber is between the lens and the back of the eye. AH is secreted in the posterior chamber by the ciliary body and flows to the anterior chamber through the pupil and exits through the trabecular meshwork into the Schlemm’s canal. Glaucoma is driven by an intraocular pressure (IOP) build up in the eye. There are a range of surgical interventions to treat glaucoma, but the key to all of these is the reduction of AH within the eye to reduce IOP.

Glaucoma drainage devices (GDDs) have been developed as a treatment and are designed to divert AH flow from the anterior chamber to an external reservoir. They have been shown to effectively reduce IOP by providing an alternate pathway for the flow of AH. GDDs however, can cause trauma to internal ocular structures, including the corneal endothelium and the iris^1^. GDDs are associated with endothelial cell damage (ECD), which may result in endothelial cell loss and corneal decompensation. An endothelial cell density below a critical point may result in a loss of corneal transparency and a reduced visual acuity. Although ECD can occur due to mechanical processes during the insertion operation, this is likely to cause a once-off point of damage and will not progress further with time. It has been speculated that mechanisms causing continued ECD are due to interrupted AH flow and circulation patterns that affect the health of the endothelial cells^2^. Additionally, clinical trials have shown that certain GDD tube parameters have caused ECD^3–7^, strongly suggesting that AH flow could be a dominant cause of ECD.

**Figure 1:**
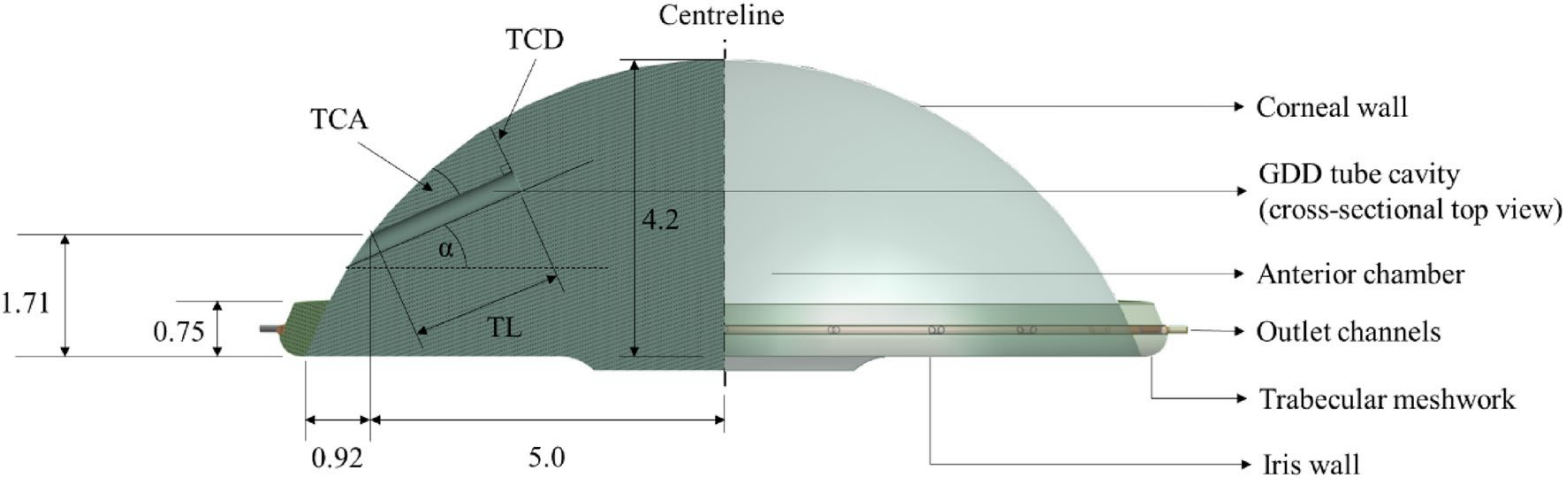
Schematic of the idealised anterior chamber with an inserted glaucoma drainage device (all dimensions in mm).

One such type of GDD is known as the Ahmed Glaucoma Valve (AGV). The insertion of an AGV is characterised by three geometric parameters: tube-cornea angle (TCA), tube-cornea distance (TCD) and tube length (TL). TCA is the angle between the inserted AGV and the corneal wall. TCD is the perpendicular distance between the AGV tip inserted and the corneal wall. TL is the inserted length of the tube. Geometrically, only two of the three are required to define the insertion orientation and the third can be derived. To ensure a successful surgery, a surgeon can control the TL and (to a certain extent) the TCA. The TCD is therefore the derived element in AGV surgery. Understanding and managing these geometric parameters will help surgeons achieve optimal results when performing AGV insertions. These and other key geometries are shown in Fig. 1. The surgical placement of the AGV and its effects on post-surgical ocular health will be the focus of this study.

A previous study by Koo et al.^6^ observed that endothelial cell density in the superior temporal quadrant was substantially lower than endothelial cell density in the inferior nasal quadrant (INQ) after AGV implantation. The study also employed both univariate and multivariate analyses to examine the relationship between tube parameters and ECD. The findings suggest that the TCD is a significant predictor of ECD, both in univariate and multivariate analyses. The other parameters examined, such as TCA, were significant in the univariate analysis but did not remain significant in the multivariate analysis. Lee et al.^3^ measured endothelial cell density pre- and post-operatively using non-contact specular microscopy to analyse cell density in patients with an AGV. They suggested that ECD is expected to occur below a TCA point of 26.70° and a TCD point of 0.90 mm. They concluded that smaller TCAs and TCDs resulted in greater ECD.

In similar clinical studies on a different GDD, the Baervedt Glaucoma Implant (BGI), Hau et al. and Tan et al.^4,7^ reported the same inverse trend between TCA and TCD with ECD. The latter reported that a mean TCD of 1.1 mm caused a central corneal endothelial cell loss of 6.20% as well as a 7.25% endothelial cell loss in the peripheral quadrant closest to the GDD tube. A larger TCD of 2.00 mm resulted in losses of 4.11% and 5.77% for the respective regions. Although these clinical studies clearly show the relationship between GDD insertion angles and position with ECD, they were conducted post-operatively and do not reveal the reasons for the observations. They are also performed on actual patients and do not consider existing medical conditions which may affect the amount of ECD.

Computational fluid dynamics (CFD) has previously been used to study various AH flow scenarios in the eye^8–17^. It has been shown that it is able to adequately predict the flow features as well as their impact on IOP. In particular, Villamarin et al.^8^ conducted a brief analysis investigating the effects of the ExPRESS V50 shunt on fluid flow features, IOP and wall shear stress (WSS). They however did not report on the changes to WSS on the corneal surface because of the insertion of the shunt nor make any indications of the incidence of ECD.

To the best of the authors’ knowledge, no comprehensive investigation has been conducted regarding the flow surrounding the AGV, which could enhance the existing clinical trials and observations. This study aims to investigate the extent of ECD due to the insertion of an AGV by studying AH flow parameters in the anterior chamber, around the AGV, in an idealised model of the eye (Fig. 1) using CFD. The AGV was selected for this study as ECD is the most common complication after AGV surgery^18^. It is important to understand the flow field in the anterior chamber when the AGV has been implanted, which may exacerbate any mechanical damage that should occur regardless of position. Furthermore, it is crucial to examine and critically analyse the effects of the various AGV tube parameters and placement on other aspects of corneal health.

## Computational methodology

**Figure 2:**
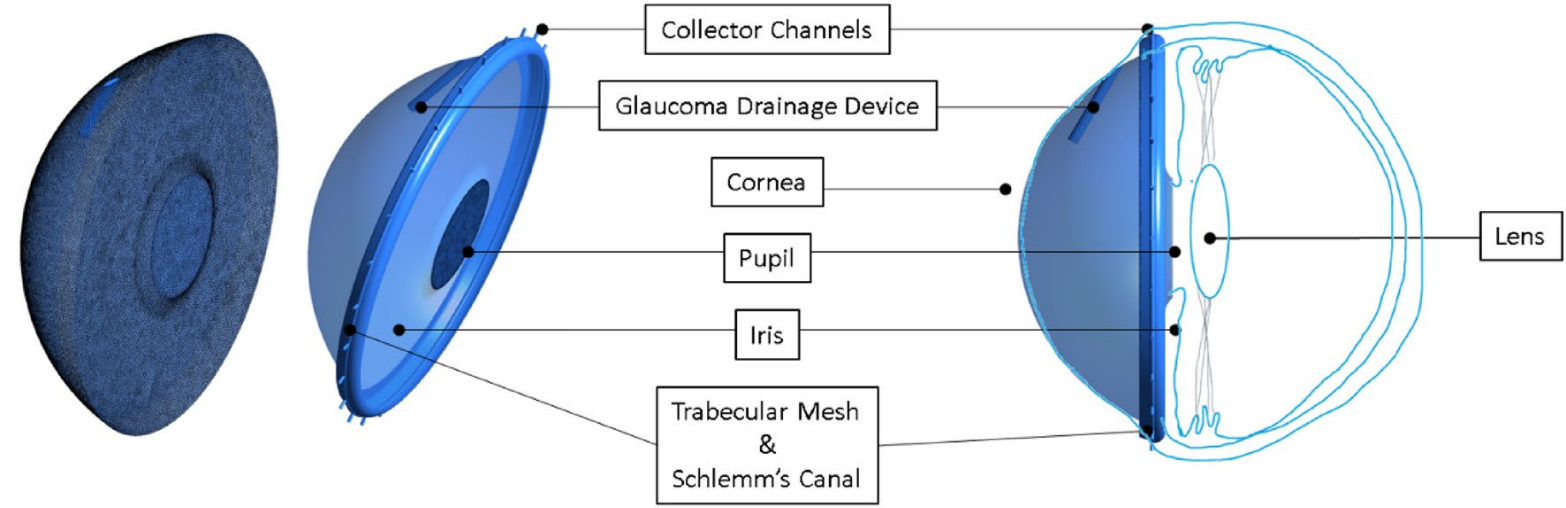
CFD domain with mesh.

A computational 3D model of an idealised anterior chamber adapted from Basson et al.^9^ with AGV insertions was used for this study. The anterior chamber model has a diameter of 11.84 mm and a depth of 4.2 mm. The model together with the final mesh is shown in Fig. 2. With a superimposed sketch of the eye’s anterior chamber for ease of reference. The AGV was inserted as shown in Fig. 1 by cutting a cylindrical shape into the flow domain. AH enters the domain (anterior chamber) through the pupil and exits through the various outlets (Collector Channels) as well as through the AGV. Inflow through the pupil is set at 5 × 10^–8^ kg/s via a mass flow inlet boundary to represent the average AH production rate in the ciliary body^8^. The outlets were set as a pressure outlet boundary at 7 mmHg. The trabecular meshwork was modelled as a porous zone with a permeability of 4.34 × 10^–16^ m^2^ to achieve glaucomatous conditions^9^. All other surfaces were modelled as a non-slip wall. The AGV was modelled as a mass flow outlet of 3 × 10^–8^ kg/s estimated using the equation from Fernandez-Vigo et. al^12^. This equation assumes the pressure drop across the device obeys the generalised Hagen-Poiseuille formula (not included here for brevity, but details are available in the paper). A functional AGV should result in an outflow that reduces the IOP from a glaucomatous condition to an acceptable non-glaucomatous value. The selected value resulted in the reduction of IOP from 27 to 14 mmHg, therefore it is appropriate.

**Figure 3:**
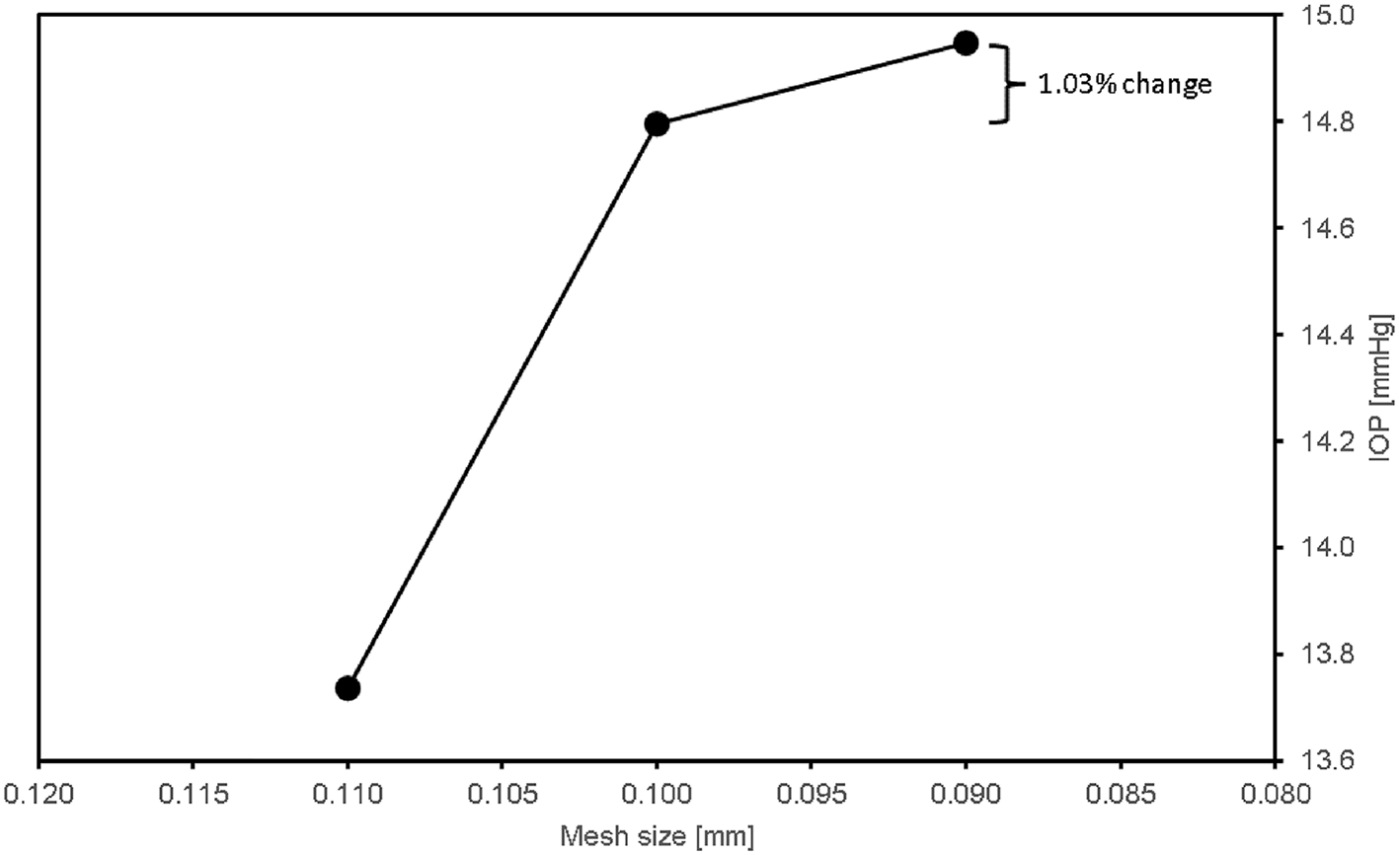
Mesh convergence study.

Mesh settings used by Basson et al.^9^ were applied as mesh independence has already been established and a further mesh convergence exercise was performed using three different mesh size settings with results given in Fig. 3. The final mesh contained approximately 6,343,151 cells. The average aspect ratio and skewness were 2.26 and 0.22 respectively, which falls into the acceptable range of 5 and 0.33. Ansys Fluent 2022 R1 was the solver employed with second-order discretisation schemes used and a convergence of 10^–4^ was reached for the residuals.

The AH flow is in the laminar regime therefore no turbulence model was used. It is assumed that AH is incompressible with fluid properties approximate to that of water (density = 1000 kg/m^3^ and dynamic viscosity = 0.001 kg/ms)^8^. AH dynamic viscosity can vary among individuals and under different physiological conditions. The present study's accuracy depends on the assumption that the chosen dynamic viscosity values are representative of the average patient population. It is also often assumed that AH flow in the eye is buoyancy-driven^19^. This assumption can be verified for the present study by determining the densimetric Richardson number, which is a dimensionless parameter used in fluid dynamics to quantify the relative importance of buoyancy forces and viscous forces in a fluid flow. It is commonly used to analyse flows with density variations, such as in the case of AH flow in the human eye. The (densimetric) Richardson number is calculated as

1 $$Ri=g^{\prime}\frac{\partial \rho /\partial z}{{\left(\partial u/\partial z\right)}^{2}}$$

where g’ is the reduced gravity $\left(g\frac{{\rho }_{1}-{\rho }_{2}}{\rho }\right)$, ρ is the density and u is the representative flow speed of AH at the pupil. The partial differential terms are estimated using the cornea depth and the iris diameter. The calculated Richardson number in both instances is in the order of 10^5^. A Ri larger than 1 indicates that buoyancy forces dominate over viscous forces. Therefore, buoyancy-driven flow is important for the present study.

The Boussinesq approximation is used to account for temperature variations in the anterior chamber^20^. This is represented in Eq. (2) and modifies the incompressible Navier–Stokes equation from Eqs. (3) to (4).

2 $$\rho ={\rho }_{0}\left[1-\beta \left(T-{T}_{ref}\right)\right]$$

3 $$\rho \left(v\cdot \nabla v\right)=-\nabla p+\mu {\cdot \nabla }^{2}v+\rho g$$

4 $$\rho v\cdot \nabla v=-\nabla p+\mu {\cdot \nabla }^{2}v+{\rho }_{0}\left[1-\beta \left(T-{T}_{ref}\right)\right]g$$

where, v is the fluid velocity, μ is the dynamic viscosity of the fluid, g is the gravitational constant, ρ_o_ is the reference density, β is the volume expansion coefficient, T is the fluid temperature, T_ref_ is the reference temperature, and p is the fluid pressure.

The range of TCA and TCD were selected from two clinical studies by Lee et al. and Tan et al.^3,7^ and ranges from 25.17° to 45.36° and 0.54 mm to 1.27 mm respectively. The tube-length (TL) was kept as a constant at 2.21 mm for this study. The silicone tube used in a AGV is designed to maintain its structural integrity and not bend when subjected to the forces typically encountered within the eye. This is crucial to ensure the device's proper function, as any bending could potentially impede fluid flow. The silicone material is selected for its rigidity and flexibility, allowing it to hold the necessary TCA without deformation. Additionally, the surgical technique involves securing the tube in place to maintain the desired angle. These factors collectively ensure that the silicone tube remains stable and does not bend, preserving the unobstructed flow of AH and facilitating the effective management of IOP in glaucoma patients. However, in the results, a calculation was performed to determine if there will be significant bending at hydrodynamic pressures experienced in the anterior chamber.

## Results and discussion

**Figure 4:**
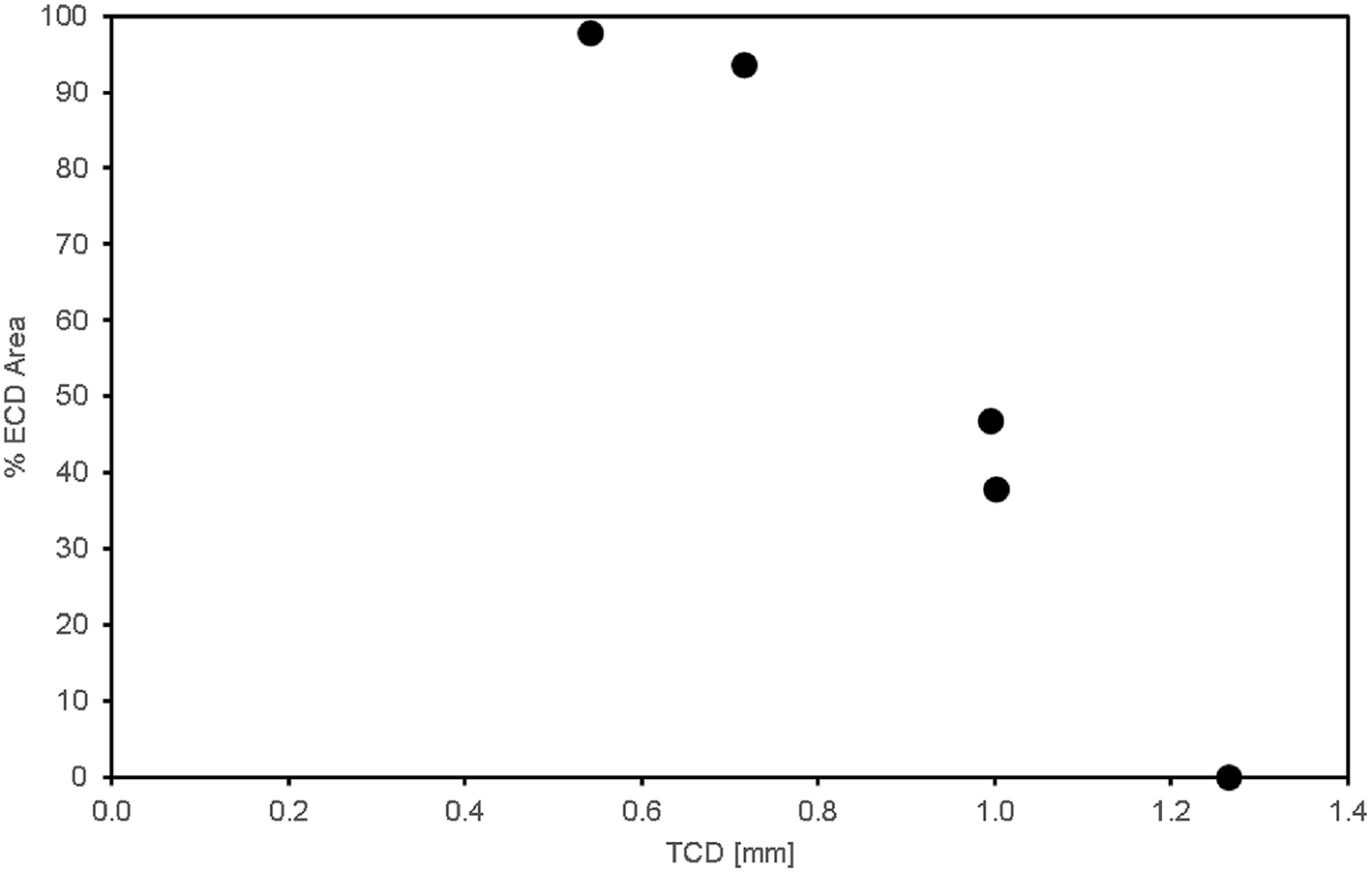
Relationship between ECD area and TCD of GDD insertion.

Kaji et al.^21^ suggested that WSS above 1 Pa is likely to cause ECD. For the remainder of this paper, regions where WSS on the cornea are 1 Pa and above, shall be termed “ECD region”. Figure 4 shows the percentage of ECD region over the entire cornea for each TCD value. One can immediately observe that there is a monotonic decrease in % ECD area with an increase in TCD, which agrees qualitatively with conclusions from previous studies^3,4,7^.

**Figure 5:**
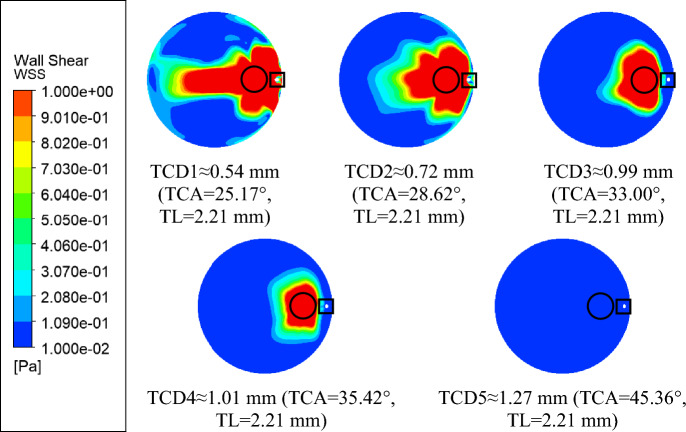
Corneal wall shear stress with different insertion parameters.

Figure 5 illustrates the ECD region with the red region denoting the ECD region where corneal WSS is higher than 1 Pa. It is located at and near the AGV insertion location and tube outlet. The insertion location is indicated with a black circle and the tube outlet with a black square. This agrees with clinical observations that ECD is greatest near the insertion location^4,5,7,22–24^. The ECD region in the TCD1 case extends from the insertion point towards the inferior nasal region in a “tail”-like projection. This is interesting because Koo et al.^6^ reported higher ECD in the inferior nasal region. They did not, however, report the insertion angles therefore direct comparison is not possible. As the TCD is increased, the overall ECD region decreases especially in the “tail”. The “tail” disappears as TCD increases above 0.99 mm. Beyond a TCD of 1.27 mm, the ECD region was non-existent.

**Figure 6:**
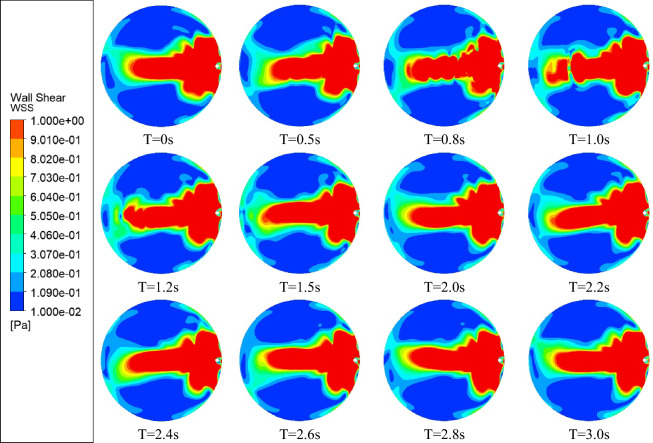
Temporal corneal wall shear stress for TCD1 ≈ 0.54 mm.

**Figure 7:**
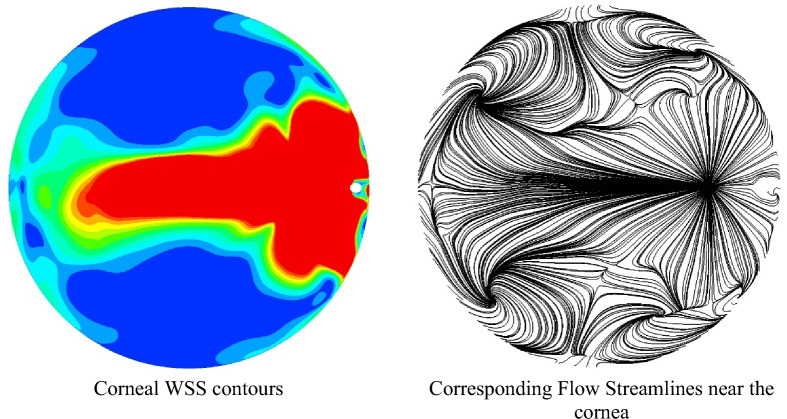
Corneal WSS and corresponding flow streamlines 0.024 mm from the cornea surface for TCD1 (T) = 1.5 s.

Transient simulations were performed with a time step of 0.01 s to investigate if the AH flow is steady. Contours of corneal WSS for TCD1 are shown in Fig. 6. Fluctuating flow was observed, and the selected time step is two orders of magnitude smaller than the smallest period of fluctuation, which happens in the TCD1 case, of approximately 1 s. Fluctuating flow is also observed in the TCD2 case, with the period larger at 4 s, but the fluctuations disappeared above TCD3. It is observed that a “wave” phenomenon occurs with the “tail” of the ECD region undergoing a repetitive pseudo-steady fluttering motion. Figure 7 shows the WSS contours and streamlines for time (T) = 1.5 s for TCD1. The AH flow streamlines at a distance 0.024 mm from the cornea surface show several swirls around the ECD region and is unlike the regular steady streamlines observed with no GDD^8,9^. There were similar observations for TCD2 but will not be presented here for brevity since they are less pronounced due to the lack of a “tail” and a longer period. This fluctuating flow results in a fluctuation of the IOP with a range of approximately 1.5 mmHg. These are an order of magnitude smaller than the IOP itself and are therefore not likely to cause perceivable palpitations in the eye. However, IOP fluctuation (and more specifically long-term fluctuation) has been identified as a risk factor for progression in several glaucoma randomised control trials^25–29^. The clinical significance of ultra-short IOP fluctuations, such as these, is not known. Tan et al.^30^ measured ultra-short fluctuations in primary angle closure eyes and identified a risk for glaucoma progression with higher amplitude-frequency ultra-short-term IOP fluctuation. This raises the possibility that IOP fluctuations induced by suboptimal AGV placement could potentially contribute to disease progression.

Aside from the minor IOP fluctuations caused by fluctuating flow, the model assumes a consistent fluid outflow via the AGV and thus a consistent post-operative IOP level when in fact, both in-vivo and in-vitro, AGV function has been shown to be inconsistent^31^. Furthermore, clinically it is common to observe a ‘hypertensive’ phase three to six weeks post-operatively that is thought to be caused by encapsulation of the bleb^32^. Raised IOP, particularly acute IOP elevation, is a recognised cause of ECD presumably from elevated WSS. Our model did not evaluate the effect of post-operative IOP elevations on WSS. It is our assumption that such WSS would be additive to that caused by the flow characteristics from the tube placement, so that even with ideal tube placement ECD could occur. In addition, Kim et al.^22^ measured ECD near the tube at 1 month, 6 months, 1 year and only found a statistically significant change from 6 months onwards (when compared to eyes without the surgery). This suggests that the impact of temporary raised IOP leading to elevated WSS and its impact on long term ECD may be less than expected.

The presence of these fluctuations in flow raises the question of whether they will induce bending or undesirable and uncontrolled oscillations and vibrations of the tube thereby making the current findings invalid. Detecting the potential for fluttering involves examining tube deflections using the hydrodynamic pressure on the tube walls. The inserted AGV was modelled as a cantilever beam, which assumes certain conditions such as small deformations, linear elasticity, and uniform material properties:

5 $$\delta =\frac{F\cdot T{L}^{4}}{8\cdot E\cdot I}$$

where $F$ is the force applied on the tube (unit width alongside the TL), $E$ is the elastic modulus of the silicone tube, and $I$ is the moment of inertia of the tube cross-section. The tube deflection δ was calculated to determine the possibility of flutter and the tube’s effect on fluid flow fluctuations. Deflection was calculated throughout one cycle of flow oscillation and is lower than 10^–5^ mm. This is many orders of magnitude smaller compared to the tube’s inner diameter of 0.305 mm suggesting that the tube does not experience significant bending or deformation.

Our findings underscore the importance of precise GDD placement, both in achieving effective pressure regulation and in minimizing IOP fluctuations, revealing the novelty of our discovery. This newfound understanding represents a significant advancement in glaucoma management, emphasizing the need for careful consideration of a GDD placement's impact on IOP dynamics. Additionally, our study highlights the critical significance of accounting for fluid force interactions during the GDD insertion procedure, particularly in relation to elevated WSS associated with specific placements. These results offer a fresh perspective on the complexities of GDD placement, promoting more precise and thoughtful surgical approaches. Ultimately, these insights have the potential to enhance patient outcomes and safety in the treatment of this vision-threatening condition.

## Conclusions

This study has used CFD to study ocular AH flow in the anterior chamber with the insertion of an AGV. The results clearly show that at certain insertion angles and locations, corneal WSS could rise to a value which may result in ECD. A TCD distance of 1.27 mm and above did not result in adverse WSS conditions that could induce ECD. A TCA angle of 45° and above had equivalent outcomes. The results correlate with previous clinical studies which advise against a TCD that is too low, i.e., below 1.27 mm. Low TCDs not only result in significant regions with elevated corneal WSS, but they also induce fluctuating flow features and fluctuating IOP which is not observed in normal eyes. There is a lack of previous reports on these specific flow characteristics, and the clinical significance of the fluctuation in IOP is yet to be established. The study did not take into account the temporary raising of IOP post-insertion of the AGV, but measurements by Kim et al.^5^, suggest this does not have a significant impact. This study shows that pathological amounts of WSS may result with certain AGV placements. Hence, it is imperative to consider the associated fluid force interactions when performing the AGV insertion procedure. Our study underscores the significance of precise AGV placement in managing IOP, minimizing fluctuations, and considering fluid force interactions, particularly in relation to elevated WSS, offering promising insights for enhancing patient outcomes and safety in glaucoma treatment.

## Data Availability

The datasets used and/or analysed during the current study are available from the corresponding author on reasonable request.
